# Land system governance shapes tick-related public and animal health risks

**DOI:** 10.1080/1747423X.2024.2330379

**Published:** 2024-04-09

**Authors:** S.O Vanwambeke, E.F Lambin, P Meyfroidt, F.A Asaaga, C Millins, B.V Purse

**Affiliations:** a Université Catholique de Louvain (UCLouvain), Earth and Life Institute (ELI), Earth and Climate Pole (ELIC), Louvain-la-Neuve, Belgium; b Fonds de la Recherche Scientifique F.R.S.-FNRS, Brussels, Belgium; c UK Centre for Ecology and Hydrology, Benson Lane, Crowmarsh Gifford, Wallingford, Oxfordshire, UK; d Institute of Infection, Veterinary and Ecological Sciences (IVES), University of Liverpool, Liverpool, UK

**Keywords:** Land use governance, health, vector-borne diseases, Kyasanur Forest Disease, Lyme disease, acaricide resistance

## Abstract

Land cover and land use have established effects on hazard and exposure to vector-borne diseases. While our understanding of the proximate and distant causes and consequences of land use decisions has evolved, the focus on the proximate effects of landscape on disease ecology remains dominant. We argue that land use governance, viewed through a land system lens, affects tick-borne disease risk. Governance affects land use trajectories and potentially shapes landscapes favourable to ticks or increases contact with ticks by structuring human-land interactions. We illustrate the role of land use legacies, trade-offs in land-use decisions, and social inequities in access to land resources, information and decision-making, with three cases: Kyasanur Forest disease in India, Lyme disease in the Outer Hebrides (Scotland), and tick acaricide resistance in cattle in Ecuador. Land use governance is key to managing the risk of tick-borne diseases, by affecting the hazard and exposure. We propose that land use governance should consider unintended consequences on infectious disease risk.

## Introduction

The relation between land cover, land use and vector-borne diseases is well established (Lambin et al., 2010; Reisen, 2010). In a suitable climate, land cover shapes hazard by providing habitat for vectors, pathogens and reservoir hosts according to their specific resource needs (Hartemink et al., 2015). Exposure is shaped through land use as human societies and domestic animals interact with the environment and come into contact with infected vectors (Lambin et al., 2010). We argue that considering the broader context of factors shaping land cover, land-use changes and human-environment interactions through land systems provides a finer understanding of long-term causal relationships. It also provides more effective, contextualised mitigation options that can be reconciled with other services expected from land.

Land system science studies the interface between social and ecological systems, with a focus on feedback loops between and within socio-ecological systems (Verburg et al., 2013). While land system science initially focused on identifying the ecological effects of land cover changes and their associated drivers, the field evolved to adopt a broader perspective on the impacts and drivers of land-use change, from individual decisions (e.g. on farms) to international trade agreements (Verburg et al., 2015). Effects of land use decisions and land-cover changes on climate, biodiversity, ecosystem services, and socio-economic changes are examined beyond their immediate and local effects through long distance consequences such as telecoupling (Liu et al., 2019; Verburg et al., 2015).

A land system approach emphasizes that maximization of all objectives associated with land use is unrealistic, and trade-offs are inherent to land use governance (Ellis et al., 2019; Meyfroidt et al., 2022). Moreover, as highlighted by Meyfroidt et al. (2022), benefits and risks from land use are unevenly distributed, and control over land resources is increasingly concentrated among fewer actors. Health risks that may be precipitated by land systems, such as pathogen spill-overs, could be mitigated by interventions that account for the ecology of infectious disease (Sokolow et al., 2019). The feasibility of land-based health interventions depends on how well they could be reconciled with other functions of land, such as food production, nature conservation and socio-economic development.

Human-nature interactions are contingent on context and affected by local value systems and may thus not be fully captured by a simple ecosystem service framework (Pascual et al., 2023). So far, ecosystem service frameworks have focused mainly on provisioning services and a few regulating services such as carbon storage and water flows, largely neglecting pest and disease vector regulation. The focus has also been on services rather than disservices (e.g. Mouchet et al., 2017; Pascual et al., 2023). As human societies face the challenge of sustaining 8 billion people while fostering other land functions such as biodiversity conservation and climate mitigation, the necessity to balance multiple priorities in local contexts has become prominent (Jones et al., 2023). Health, however, has remained largely excluded from this discussion. By contrast, the field of disease ecology considers land use as an important driver of human spill-over, but often overlooks the complexity of land systems as social-ecological systems. This paper aims to enhance the recognition of health as a function of land systems and as a meaningful objective of land use governance, alongside food security, climate change mitigation, biodiversity conservation, etc. It also illustrates the importance of considering complex land system dynamics and land use governance in disease ecology, using the example of tick-borne diseases (TBD).

**Figure 1.**
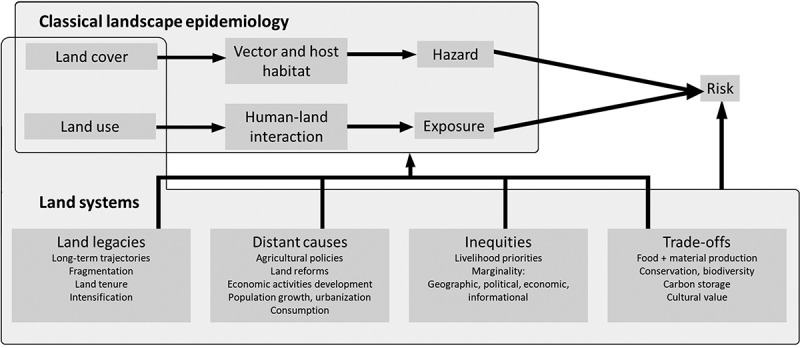
Summary of the proposed relations between land systems and vector-borne disease ecology.

Several dimensions of land systems are relevant to health in the context of TBD (Figure 1):

- Land systems result from long-term interactions between human societies and the environment. Landscapes today result from decisions made over decades or centuries (Ellis, 2021).
- Both proximate (i.e. immediate actions at the local level) and distant, underlying causes of land systems change (i.e. fundamental societal processes such as population growth or policies) must be understood to interpret past patterns and steer future land use decisions (Geist & Lambin, 2002; Meyfroidt et al., 2018).
- Multiple stakeholders hold different sets of values affecting decisions relevant to land. Their agency in that process may be variable (Ellis et al., 2019; Pascual et al., 2017). Better recognition of the many stakeholders of land use and of the diverse values they hold could foster more equitable land use governance and avoid unintended consequences of land use decisions. This is critical for communities that are marginal to land use decisions and health systems and whose exposure to environmentally acquired pathogens can also be greater.
- Most land is used for multiple purposes or fulfills multiple functions – e.g. producing food and wood, storing carbon, contributing to conservation and providing access to green spaces (Meyfroidt et al., 2022). This means that land management to minimize health risk can only be one amongst several objectives. Moreover, exposure and adaptation may be linked to diverse livelihood priorities associated with land use (Asaaga et al., 2022).
- Because land serves multiple purposes which may or may not constitute priorities for various stakeholders, trade-offs are often inevitable in land use decisions. Accounting for health adds a critical dimension to achieve the UN Sustainable Development Goals (Jones et al., 2023).
- The importance of inequities is becoming increasingly visible in land systems. The concentration of land in a smaller pool of owners or managers is accelerating, while, large rural populations remain trapped in poverty, lacking access to infrastructure and services (Meyfroidt et al., 2022). Health is subject to strong inequities, both in exposure to risk and access to control, treatment or other adaptive options. Vulnerable communities are often remote from health systems, political systems and land use decision-making.

We first briefly review existing work on land and health and illustrate it with the case of Lyme disease. We then present three case studies that highlight the role of land use governance in shaping tick-borne disease risks: Kyasanur Forest Disease in India, Lyme disease in the Outer Hebrides (Scotland) and tick acaricide resistance in Ecuador. Finally, we discuss how land use governance frameworks should consider health disbenefits and co-benefits, and conversely, how health actors could tap the potential of land use governance to mitigate health risks.

## Background

### The health dimension of land

Disease ecologists have long been interested in the proximate relationship between vector-borne disease risk and land cover (May, 1952; Pavlovsky, 1966; Sorre, 1933). The advent of remote sensing and geographical information systems within landscape ecology enabled detailed monitoring of ecosystems and led to an intensified research focus on links between vector and host habitats and landscape features (Beck & Lobitz, 2000; Kitron, 1998; Reisen, 2010). In landscape epidemiology, humans were initially considered as susceptible hosts, unevenly exposed to infectious vectors (Patz et al., 2000). Human activities were later accounted for by distinguishing between land cover and land use (Lambin et al., 2010; Murray & Daszak, 2013) and linking human exposure to behaviours and activities in different land uses (Zeimes et al., 2014), incorporating spatial complexity in landscape structure and associated overlapping distributions of hosts and vectors (Diuk-Wasser et al., 2020; Li et al., 2012, 2019). Land ownership was shown to moderate human access and affect vector abundance by altering landscape structure (Lambin et al., 2010). Inputs from functional ecology highlighted how host-vector-pathogen interactions may be predicted from their resource use in the landscape and suggested that land management may offer effective risk management options (Hartemink et al., 2015).

The focus on proximate relations to land in the case of TBD is well illustrated by the case of Lyme disease in Europe and North America. The tick *Ixodes ricinus*, in Europe, and *Ixodes scapularis* in Eastern North America, vectors of *Borrelia burgdorferi*, the pathogenic agent of Lyme disease in these regions, are predominantly found in temperate forests or at their edge. Ticks feed on a large diversity of hosts, ranging from mammals to birds and reptiles (Gray et al., 2021; Kahl & Gray, 2023). *I. ricinus* and *I. scapularis* have a three-host lifecycle with periods of weeks to months of developmental dormancy between life stages (called diapause) in the environment, which heavily determines which habitats are suitable. Deciduous forests with dense undergrowth, thick leaf litter and abundant blood meal hosts have drawn much attention over the years as tick habitats. Epidemiological data, however, indicate that ecological resources for ticks can be found in other places as well, including peri-urban habitats, urban parks, gardens, grassland and moorlands (Hansford et al., 2023; Mathews-Martin et al., 2020; Medlock et al., 2020; Millins et al., 2021; Rizzoli et al., 2014). This observation suggests a more nuanced association of ticks with land cover and a strong role of land use in shaping risk (Vanwambeke & Schimit, 2021).

The relationship between landscape, tick hosts and infectious tick density is complex. Ticks feed opportunistically on a broad range of hosts, each with different associations to land use and landscape features (Hartemink et al., 2015). Hosts may have various physical (e.g. grooming) and immunological defences against ticks. Some hosts efficiently transmit pathogens to ticks (termed transmission hosts). Others play an important role in feeding adult female ticks (termed tick reproduction hosts) and thus affect tick fecundity, abundance and hazard (Mysterud et al., 2021; VanAcker et al., 2019). Landscapes shape the distribution and density of reproduction and reservoir hosts, resulting in spatially varying tick abundance and Lyme disease hazard. These associations between TBD risk and host ecology, as mediated by landscape, can be scale and context dependent (Gandy et al., 2022; Wood & Lafferty, 2013). The role of biodiversity in driving Lyme disease risk has been heavily debated (Millins et al., 2017; Randolph & Dobson, 2012), but the role of forest management has been comparatively less studied (but see Tack et al., 2012, 2013).

Finally, human-environment interactions shape exposure, a key component of human incidence of diseases transmitted by *I. ricinus* and *I. scapularis*. Because these ticks can only move a few centimetres in the environment (unless transported by a host), exposure relies on humans entering tick-infested habitats. Human disease incidence depends on the type of activity (e.g. professional or recreational), the intensity of exposure to tick-infested habitats (De Keukeleire et al., 2017, 2018; Jore et al., 2020), and attractivity of landscape features, even when tick abundance is low (Van Gestel et al., 2021; Zeimes et al., 2014). Despite the lack of effective human vaccines and some research on reproductive host management for tick control (Gilbert et al., 2012), tick control based on land management has scarcely been investigated. Fire (Gleim et al., 2019; Padgett et al., 2009) and vegetation removal have been tested (Allan et al., 2010; Conte et al., 2021; Tack et al., 2013), but their compatibility with other land functions has not been examined. No effective land-based tick control strategy has so far been identified. Disease control rests largely on awareness raising and individuals preventing bites (Eisen, 2021). Forest and wildlife management, as well as peri-urbanisation, in many areas of Europe and North America over the past 50 years, may have fostered tick and host populations and their proximity to humans. Most studies have considered the landscape in a static fashion, neglecting to assess how landscape transformation may have influenced risk (but see the case of tick-borne encephalitis in the Baltics (Randolph & Šumilo, 2007; Vanwambeke et al., 2010). This well-studied system underlines the strong but complex connections between landscape and TBD risk. It illustrates the limited understanding of broader aspects of land management, land use histories, and potentially conflicting land functions, and how these are embedded in and influenced by land use governance.

### Health and land in the context of global change

One Health has contributed greatly to bring attention to the multiple dimensions of health, including as a feature of socio-ecosystems (Zinsstag et al., 2011). The acceleration of global change (Richardson et al., 2023) and the evolving burden of zoonotic and vector-borne diseases add a sense of urgency to the need to mainstream One Health in various policies (Zinsstag et al., 2023). Discourses have changed from a perspective of ‘unhealthy landscapes’ (Patz et al., 2004) towards a more positive view of environment’s contribution to health with the concept of ‘landscape immunity’, calling for land management protective of health (Reaser et al., 2022). In this view, maintaining the immune function of wild species, to minimize pathogen prevalence and shedding, and a high proportion and diversity of non-competent hosts within communities (known as the dilution effect) is key to preventing microbes in the wild from becoming pathogenic threats to (human) health. However, health threats associated with land do not only occur as zoonotic pathogen spill-overs resulting from ecosystem encroachment (Plowright et al., 2021) or intensification (IPBES, 2020). Consideration of longer-term factors shaping landscapes and vulnerability and adaptive capacity of communities is also important to proactively address health in the context of land use decisions, especially for marginalised populations. For the definition of hazard, exposure, vulnerability and adaptive capacity, we refer to the IPCC glossary (Intergovernmental Panel on Climate Change IPCC, 2022).

**Figure 2.**
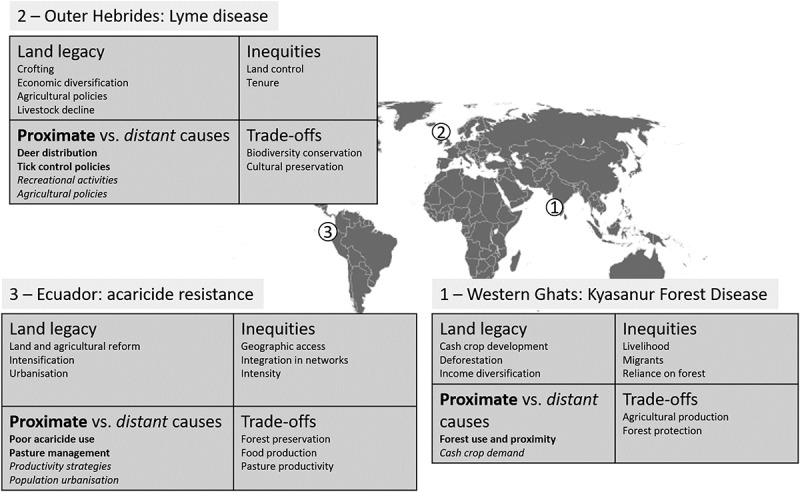
Location of three case studies and summary of land system dimensions.

Land use governance has the potential to prevent and mitigate human and animal health risks associated with land use change. We argue that land management decisions must account for unintended consequences for health that arise from diverse land use priorities and complex human-interactions. Below, we present three case studies from contrasting global settings (Figure 2) to illustrate how, apart from the land cover/hazard and land use/exposure associations, the broader dimensions of land systems could help understand emerging public and animal health risks early in their development and identify the most vulnerable communities.

## Case studies

### Kyasanur forest disease in India: when forest clearing, population growth, and forest use collide

Kyasanur Forest Disease (KFD) is a debilitating and potentially fatal haemorrhagic human disease, caused by the tick-borne Kyasanur Forest Disease Virus (KFDV; genus Flavivirus), emerging across the degraded Western Ghats forest ecosystem in South India. Humans contract KFD when bitten by an infected tick, but are incidental or ‘dead-end’ hosts for KFDV. KFDV is thought to be maintained in silent forest transmission cycles between ticks and wide-ranging wildlife transmission hosts including wild rodents and shrews, monkeys and birds (Pattnaik, 2006; Work et al., 1959). Domestic cattle are thought to amplify tick populations through their importance as a blood meal host. KFD has been largely managed through human centred interventions, including an unlicensed vaccine, awareness raising, personal protective measures including acaricides applied to cattle, people and habitat. The evidence base for and uptake of these measures is poor (Burthe et al., 2021).

KFD outbreaks first emerged in the 1950s and were attributed to deforestation for human settlement and roads (Work et al., 1959). It has remained restricted to focal areas of Karnataka State for decades. Since 2014, human cases have been detected in four neighbouring states (Tamil Nadu, Kerala, Goa and Maharashtra) (Chakraborty et al., 2019). Migrant agricultural labourers in plantations, people involved in cultivation and grazing of cattle in and around forests, and tribal groups who gather non-timber forest products have all been affected by recent outbreaks in Maharashtra and Goa (Kasabi et al., 2013; Patil et al., 2017). It appears urgent to delineate the land system conditions and socio-ecological processes, including historical land use transitions, that have precipitated human disease impacts.

Large-scale conversion of the tropical humid forests of the Western Ghats to plantations (initially tea, coffee and cardamom) began in the 19th century as colonial enterprises. It was continued post-independence by State actors, private developers, and international development agencies (Krishnan, 2009). An estimated 35% (33,579 km2) of natural forest was lost in the Western Ghats from the 1920s to 2013 and transformed mostly to plantations, agriculture and scrub (Reddy et al., 2016). Following stricter forest regulations post-1980s, many of the Western Ghats forest-agricultural areas were designated as protected forest reserves (Bawa et al., 2007). Several strands of correlative evidence link KFD spill-over to this large-scale forest conversion. Large outbreaks in the 1970s and 1980s followed the replacement of evergreen forests with cashew nut plantations under international development projects (Nichter, 1987), underlining the role of distant factors.

In parallel, human populations have grown around forests and forest protected areas, with an estimated 50 million people currently living in the Western Ghats (Kasturirangan et al., 2013). This has led to increased demand for housing, roads, agricultural commodities and paddy cultivation for subsistence, increased human–wildlife conflict and highly contested land and resources (Sreeja et al., 2021). More recent institutional drivers of land-use change include state-level incentives for smallholders to combine arecanut plantations with mixed crops and dairying within an integrated system. This would bolster income security and meet increasing demand for arecanut (Sujatha & Bhat, 2015; Tigari & Rajamma, 2019).

Recent work (2014–2018) on climate, social, host and landscape patterns associated human outbreaks, with diverse forest-agricultural mosaics containing high cover of moist evergreen forest and plantation (including arecanut), low cover of dry deciduous forests, and high densities of indigenous cattle (Purse et al., 2020). This confirmed the role of long-term landscape changes. The socio-ecological mechanisms that have rendered diverse social groups vulnerable to varying degrees to KFD in degraded, multi-use forest landscape mosaics of the Western Ghats have been studied through co-located social and ecological surveys, landscape analysis and epidemiological modelling along forest-agriculture gradients (Asaaga et al., 2021, 2023; Purse et al., 2023). Within this land system, vulnerability depends not only on variable tick hazard between different habitats in the mosaic but also on livelihood practices and priorities that influence exposure across these habitat types as well as the geographical and social marginalisation of these communities (Asaaga et al., 2022, 2023).

Considering first how forest-agriculture mosaic shape hazard, spill-over has been facilitated by a landscape of dense interfaces between tropical evergreen and deciduous forests, cultivation such as paddy, and scrub habitat between villages. Tick surveys have indicated that, although tick abundance is highest in forests, the risk of human exposure to infected ticks extends to forest edges, plantations and surrounding houses and gardens (Purse et al., 2023). Moreover, the collection of dry leaf litter from forests for various household purposes bring infected ticks into homes (MonkeyFeverRisk unpublished data). Tick populations, grazing cattle, wildlife transmission hosts and susceptible humans thus encounter each other in many landscape units (Despommier et al., 2007; Purse et al., 2020). Further ecological research is needed to understand how different wildlife hosts and cattle contribute to maintaining transmission and tick populations across different habitats and under different extents of forest transition (Burthe et al., 2021). However, mechanistic modelling across habitats in the forest-agricultural mosaic indicates that small mammals are likely to be much more important than monkeys and birds in maintaining KFDV transmission and should be accounted for in management (Hassall et al., 2023).

The burden of KFD is unevenly distributed. According to household surveys and interviews in affected areas (see Asaaga et al., 2021, 2023 for methods), smallholders from households that were low caste, land-poor, below the poverty line, headed by an elderly person, with poor access to disease information perceived themselves as most vulnerable to KFD-related impacts. These groups have a significantly higher risk of tick exposure and KFDV infection due to their occupations in forests, plantations, and crop fields within the forest-agricultural mosaic. In communities affected since 2018, in Sagara Taluk, Shivamogga District, for example, plantation workers (46%), housewives (18%) and farmers (15%) constituted most cases (*n* = 39) and 65% of respondents had visited forests or plantations within 10 days prior to diagnosis (*n* = 23). These communities included people that had been resettled from protected forest areas elsewhere in Karnataka State, highlighting how displaced communities can face novel land-mediated health risks to which they are not prepared. Place-based factors also affected vulnerability, including proximity to private hospitals and main roads. In terms of marginalization from knowledge networks, there was generally limited awareness of KFD in the surveyed communities, even in communities with recent cases, with about two-thirds of survey respondents not employing any strategies to prevent tick bites. Households with better access to information were more likely to take adaptive actions, which included vaccination, avoiding visits to forests, wearing protective clothing and footwear, applying tick repellent and attempting to diversify their income away from forest-related sources (Asaaga et al., 2021). Barriers to taking these actions, aside from lack of information, included low efficacy of the current vaccine and tick repellents, mistrust, religious or cultural or livelihood concerns.

Vulnerability is thus socially differentiated and strongly tied to livelihood priorities, place-based factors and socio-economic and cultural marginalisation. This finding is important to integrate within a land systems framework for health, to allow for context-specific prioritisation and targeting of interventions. Homogenous labelling of smallholders as ‘vulnerable’ could compromise or favour certain intervention pathways, which might threaten or worsen the already precarious livelihoods of certain social groups (e.g. tribal forest-dependent households) with weaker bargaining power or influence (Asaaga et al., 2023). Thus, existing policies banning people from entering forests in response to KFD outbreaks are likely to have adverse effects on their livelihoods and wellbeing. Intervention, including on land use (e.g. forest bans, diversification of livelihood activities) should be relevant to local livelihood and evaluated against potential negative consequences. Designing incentives to diversify livelihoods and policies addressing forest use also requires being mindful of the historical conflicts surrounding conservation and forest protection in these regions.

### Lyme disease in the Outer Hebrides: preserving a traditional way of life, fulfilling new land functions

Tick-borne diseases have emerged on the Outer Hebrides archipelago on the west coast of Scotland in the last decade as a significant concern for human and animal health, with the emergence of Lyme disease (Millins et al., 2021) and frequent cases of TBD in livestock (Jeffries et al., 2014). Crofting is the predominate land-use and the foundation of the culture and way of life. It is a system of small agricultural holdings unique to the Scottish Highlands and Islands, and requires tenants to reside in the vicinity of the croft (undefined). Populations live in dispersed rural townships in mainly coastal areas. The economy relies mainly on primary industries and the public sector, with traditional agricultural and fishing activities providing a secondary income for the majority. Surveys distributed to residents in the Outer Hebrides to understand the drivers of tick bite exposure support a changing distribution of ticks, from a mainly moorland distribution distant from townships and crofts in past decades to a more generalized distribution across the islands in both moorland and grassland habitats close to people’s homes in the present day (Millins et al., 2021). Contemporary ecological studies show that tick density and hazard from Lyme disease on islands of Uist in the Outer Hebrides, which are most affected by Lyme disease, are similar across the main land cover types, with grassland and gardens around crofts having similar tick densities and infection rate to moorland and peatland areas (Millins et al., 2021).

Landscapes in the Hebrides have been shaped by crofting over centuries. Notable land reforms were introduced in the 19^th^ century, including with the Crofters Holdings Act (1886) introduced to give crofters the security of land tenure and rights and responsibilities. Over the last century, crofting has changed from being a full-time job to provide sufficient food for a family to survive on, to being a traditional activity and way of life in the modern day. With an average size of approximately 5 ha, crofts cannot constitute a sole source of household income. Crofting practices have been passed down through generations and are now most commonly regarded as part of the cultural identity and as a responsibility to look after the land, habitats, and wildlife. The shift away from their primary source of subsistence, led to marked changes in land and livestock management practices over the last 40 years.

Large changes in deer and livestock density, movements and management in recent decades likely have had direct effects on tick abundance through their role in feeding adult female ticks (Mysterud et al., 2016), both positive and negative ones. Since 1980, there has been an approximate 50% reduction in the number of crofts that keep livestock, and numbers of sheep and cattle have halved overall (Scottish Government, 2023). Livestock husbandry practices have changed over the same period, with sheep now kept closer to crofts, compared to higher grazing intensities on moorland hill ground in the past. Sheep are now mainly stocked on grassland around crofts and on coastal grassland. Larger meat-producing breeds of sheep are often preferred by crofters compared to traditional native breeds which are smaller but more resistant to ticks and tick-borne pathogens. The changes in sheep number and management have been associated with multiple factors. With crofting a part-time activity, time may be insufficient to use more distant common grazing areas and keep sheep away from the croft.

Besides number and distribution, other changes in sheep management may have affected tick abundance. Treatment of sheep for ectoparasites changed in the 1980s when compulsory sheep dipping for sheep scab was deregulated and replaced by a range of voluntary acaricide management practices (French et al., 1999). The impact of this on tick dynamics is not well understood ecologically but is linked by residents and crofters to an increase in problems with ticks. However, changes in tick host abundance and distribution cannot be understood solely by looking at livestock. The reduction in livestock numbers and changes in agricultural practices has co-occurred with the introduction of red deer to the South Uist in 1975 for stalking and expansion of red deer population on other islands (Fletcher, 2000). Increasing encroachment of red deer from moorland onto croftland and coastal grassland in the last decade as deer move into peri-domestic areas to feed on crops, gardens and livestock grazing areas has been associated by crofters and residents with increasing issues from ticks (Millins et al., 2021). Similar trends in livestock numbers, management and deer populations and interface with deer and humans are likely to be occurring more widely in rural areas of the UK and northern Europe (Millins et al., 2017; Pepper et al., 2020).

Distant causes of agricultural and environmental policies also warrant examination. Government rural payments have changed with payments under the European Common Agricultural Policy being gradually replaced by different iterations of agri-environmental schemes rewarding nature-friendly farming schemes (NatureScot, n.d.). Crofting agricultural practices are considered of high nature value with low intensity and creation of a mosaic of cropping and semi-natural grasslands, which is highly beneficial for wildlife and biodiversity (NatureScot, 2023). Trade-offs between agri-environmental schemes to promote biodiversity and their unintended consequences for ticks and tick-borne diseases are poorly understood and warrant further study.

Future challenges lie in understanding how effective a range of potential environmental, human and livestock centred interventions are to reduce tick and TBD hazard and to reduce exposure of humans and livestock, whether these interventions are acceptable to all stakeholders, what the trade-offs are, and who bears the costs and who receives benefits. Future interventions designed to reduce the density of infected ticks in the environment, by targeting hosts of adult ticks through livestock acaricide treatment or deer culling or fencing are challenging for several reasons. Firstly, there are time lags of several years between management and changes in tick hazard, which make empirical ecological studies to test the effectiveness of interventions difficult to complete. Secondly, tick hazard and the risk of TBD is influenced by connectivity and movement of livestock and deer between different habitats with contrasting land uses and stakeholder priorities, i.e. moorland being predominately grazed by deer which are managed for stalking by estates, and croft grassland predominately grazed by cattle and sheep by crofters.

### Acaricide resistance in cattle ticks in Ecuador: access to land and productivity improvements

Cattle ranching in Ecuador is currently facing an important challenge with acaricide resistance in *Rhipicephalus microplus*, a tick infesting bovines in many herds throughout the country (Paucar et al., 2022; Pérez-Otáñez et al., 2023), and transmitting pathogens such as *Anaplasma marginale* (Guarnizo et al., 2020; Maya-Delgado et al., 2020). Today, it is estimated that ticks in as much as 60% of the farms have some degree of resistance to at least one acaricide and that resistance to multiple active substances is common (Pérez-Otáñez et al., 2024). Farmers’ practices are suspected to be a major proximate cause of acaricide resistance build up (Pérez-Otáñez et al., 2024). Inappropriate rotation of active substances is frequent, possibly caused by their marketing under different brand names (Paucar-Quishpe et al., 2023). Poor application and dosing are also frequent. Cattle are economically important in Ecuador, providing a livelihood for their owners and animal-source proteins to a growing and increasingly urban population. Understanding the historical development of cattle farming allows us to formulate hypotheses on distant causes for acaricide resistance.

The current landscape of cattle farming is rooted in the development of commercial farming over decades. Cattle have played a prominent role in shaping landscapes through deforestation for pastures and as a means to secure land tenure (Grijalva-Olmedo et al., 2010; Wood & Porro, 2002). Since the independence of Ecuador, major developments in agricultural activities have involved investment in cash crops such as banana and cocoa. Throughout the late 19th and early 20th century, this created an extremely unequal distribution of land, with many landless farm workers or small tenants dependent on labour on plantations (Grijalva-Olmedo et al., 2010). When market crops crashed due to diseases or international market price drops, the uneven distribution of land and income left many people in great difficulty and created a pressing need for land reform. In the second half of the 20th century, agricultural and land reforms supported land colonization on the eastern flank of the Andes and agriculture modernisation, with the support of cheap credit provided by oil revenues (Grijalva et al., 2004). Conditions for cropping are harsh in the Andes, where soil depletes fast, temperatures are low and sunlight scarce. Pioneer crops were soon replaced by pastures used for grazing cattle. For livestock-based farms, genetic improvement of stock and use of machinery were at the heart of the modernization. Early settlers were able to take advantage of the flattest, most accessible land near the road network, expanding to open up oil drilling regions. Later, settlers had to use more distant, steeper and less accessible plots. Cattle stock grew very rapidly during the 1970s and through the 1980s (FAOSTAT, 2023). Ecuador’s population urbanized rapidly, to become predominantly urban in the 1980s, generating an increased demand for animal-sourced food (FAOSTAT, 2023). Cattle stock stabilized at the end of the century, as milk, and more recently meat consumption stabilized and credit became less accessible.

Since the early 2000s, concerns for the environment and for cattle productivity encouraged the search for improved pasture management practices. More nutritive grass species, faster pasture rotation favouring plant growth and higher grazing load have all contributed to marginal land abandonment and, in some areas, slowing down deforestation (although it continues in the coastal region and Amazon basin). The development of the dairy industry and chains of commercialization also made land close to the road network more attractive. These changes in farm management may have acted as proximate causes of greater tick pressure, by fostering greater cattle loads and higher frequencies of cattle returning to a pasture. Because *R. microplus* larvae are sensitive to weather and microclimate at grass level, when they are off the host (de Barros et al., 2017; Leal et al., 2018), leaving pastures to rest, and thus tick larvae to die over time, may lead to a lower tick load on the grass, but is unfavourable to fodder grass growth and nutritional value.

Today, the productivity of cattle farming in Ecuador remains low in most cases. Much effort is dedicated to genetic improvement, often not accounting for the adaptation (or lack thereof) of breeds to the harsh environmental conditions encountered in Ecuador, including the strong pressure of ticks. With many herd owners raising cattle as a secondary activity, veterinary health literacy is poor, particularly concerning ticks.

The emergence of cattle farming stemmed from land and agricultural reforms aiming, among other objectives, to foster a more even distribution of land, increase production and improve productivity. More recently, objectives to preserve forested land have also emerged. Past reforms created numerous small holdings that have not consolidated or expanded over time. Most farms remain extensive and struggling with managing herd health issues (as assessed by tick burden and acaricide resistance). The attractivity of better land situated close to the road network, along with evolving pasture management, have also created a favourable context for ticks to persist.

## Discussion

Our case studies show how land use decisions made over long periods of time have the unintended consequences of a heavy burden of tick-related issues, pathogens in two cases, and acaricide resistance in one (Figure 2). By examining our cases through the land systems lens, we highlight key mechanisms that link land system dynamics and their governance to disease risks. These provide important lessons for a successful implementation of One Health policies. Our findings suggest that health-related consequences of land use are often missing among current trade-offs in land system governance.

### Understanding tick-related health issues under a land system perspective

#### Land use legacies: systems result from sustained interaction

Examining land use legacies was useful in each case for understanding the emergence of TBD. In India, the current forest mosaic landscape results from century-old policies of cash crop development. Contemporary policies of income diversification in the context of a densely populated area have increased the reliance on forest fragments encroached over decades, bringing vulnerable human populations into tick-infested environments. In Scotland, evolution of crofting, agricultural and animal health policies, and the development of recreational activities over several decades may have all contributed to high tick densities in the vicinity of houses. Changing livestock population and distribution, and the introduction of deer and its broad distribution on the Island may have changed the feeding success and redistribution of ticks over the island. In Ecuador, the current situation reflects multiple land and agricultural reforms, along with urbanisation. Policies have not only supported the development of cattle farming but also attempted to improve productivity, with steep increases in stock densities over the second half of the 20^th^ century, concomitant to a search for improved pasture productivity, and increasing concern for forest preservation. All three cases studies suggest that the tick-related health issues emerged over long periods as landscapes became more favourable for ticks and tick-host interactions as a result of land use decisions. This had been observed also in the context of the surge in tick-borne encephalitis in the Baltic states during the 1990s after massive farmland abandonment and changes in land use (Randolph et al., 2001; Vanwambeke et al., 2010). Livestock feature as key reproductive hosts for ticks and livestock keeping as an important livelihood activity promoting exposure in all case studies. In these very different settings, while livestock is not necessarily a primary aspect of food security and livelihood, it is at a crucial interface between conservation, intensification and human and animal health (Jones et al., 2013; Vanwambeke et al., 2019).

#### Proximate and distant causes affect land use and thus its health consequences

All three case studies feature national or regional land and agricultural reforms and policies, as well as changes in population number or composition. Commercial agricultural development or, in the case of Scotland, the development of rural commercial activities (deer stalking) created economic opportunities and contributed to the underlying causes of health issues. Proximate causes include human behaviours that directly affect tick-related health issues, such as poor acaricide treatment practices in Ecuador or interruption of treatment in Scotland. Proximate causes also include pasture management (Ecuador) or grazing practices (India and Scotland). In Ecuador, a focus on productivity, through grassland productivity and animal genetics, may have gradually produced a system favourable to heavy tick burdens. Tick abundance could be thus an unintended consequence of the development of cattle rearing as a commercial activity. Given that the health context is influenced by direct and indirect factors, ranging from human behaviours to land management practices to policies and broader socio-economic changes, addressing health-related issues requires interventions at these different levels. As advocated by One Health, it requires mainstreaming health in all policies.

#### Inequities between various stakeholders result in inequities in exposure

The case study on KFD in India points to strong power and risk asymmetries between stakeholders, with smallholder communities often bearing the heaviest health burden and having the least access to decision-making, information or support. In Scotland, crofters must share the landscape with larger landowners with very different priorities and resources. There are large power imbalances between landowners and tenants. In Ecuador, farms differ in intensity (herd size, mechanisation) (Paucar et al., 2022; Paucar-Quishpe et al., 2023) and in their degree of integration into commercial networks and of geographic access to infrastructure and services. Acaricide resistance is associated with farming practices, with different levels of intensification, but the link between farm profiles and trajectories, practices and resistance is still hypothetical and warrants further investigation. Interventions that are insufficiently contextualized risk being ineffective or inefficient or causing unintended side effects (Asaaga et al., 2021, 2023). Our three case studies show that avoiding this requires considering all dimensions of vulnerability and their connections to ticks exposure and adaptive strategies. Beyond these local stakeholders, decision-makers in agriculture, nature conservation, and human and animal health also intervene in land use governance at various levels. By exploring connections between land use, health policies and decision-making, and risk, an integrated land system approach reveals that vulnerability and adaptive capacity to vector-borne diseases is socially differentiated among marginalised populations and linked to precarious livelihood strategies (Asaaga et al., 2022).

#### Land use governance always involves multiple functions and trade-offs

Landscapes support multiple functions in our case studies: commercial food production, subsistence livelihoods and the attached cultural values, rural economic development, and forest or moorland conservation. Because of the multiplicity of land functions and stakeholders’ diverse priorities, strategies and claims, trade-offs are unavoidable. In some instances, multiple functions have been deemed compatible, such as in the case of crofts providing livelihoods, preserving a tradition, and fostering local biodiversity. In other cases, functions need to operate in contexts characterized by strong competition between agricultural production (expanding cropland or pastures), human settlement, and forest conservation. Changing land use distributions over time makes the reconciliation of various functions challenging in some contexts. This can lead to ticks, people and cattle overlapping, favouring spill-overs, as in the case of KFD in India. In densely populated India, where forests have been heavily degraded and are now interspersed in a mosaic landscape with numerous interfaces, the livelihood of large rural populations relies on forest resources. These populations are exposed to ticks that are part of the natural forest ecosystem. Forest bans would disproportionately affect these communities and lead to loss of income, food and fuel. In Ecuador, geographic accessibility to marketing networks and transformation infrastructure (e.g. dairy product treatment plants) is making more distant land less desirable (Porro et al., 2012) and may preserve or restore forest in less strategically located lands (Rudel et al., 2002). Improved forage productivity and nutritional values comes at the expense of higher tick burden on pastures, as would a strategy concentrating dairy production along transportation infrastructure. Crofting in modern day Scotland is valued culturally and for its biodiversity benefits but has changed substantially, becoming a secondary activity, with fewer livestock being maintained in areas distant from residences. New economic opportunities have arisen that use land in a very different way, with deer as wild animals that roam free but encroach on other land uses. Acknowledging how trade-offs play out in various contexts is a first step towards building contextualized solutions that balance the values of a wide range of stakeholders (Jones et al., 2023; Meyfroidt et al., 2022; Verburg et al., 2015).

### Integrating health into land system governance

By applying the land system approach, we can understand health risks such as vector-borne diseases as an unequally distributed burden from land use, often linked to contested land use and land tenure (Meyfroidt et al., 2022) and concentrated among marginalised communities (Chaves et al., 2023). Epidemiological studies have previously shown the role of socio-economic marginalisation in driving vector-borne disease patterns by incorporating poverty or social marginalisation indices alongside landscape metrics as predictors of disease occurrence or severity (e.g. Chaves et al., 2008). Further studies have highlighted poverty and geographical remoteness as key drivers of vulnerability to vector-borne diseases (Aagaard-Hansen & Chaignat, 2010; Kar et al., 2014). Multiple aspects of marginality can interact to underpin vulnerability and adaptive capacity to vector-borne diseases (Asaaga et al., 2022; Bardosh et al., 2017; Dzingirai et al., 2017), with communities with low socio-economic status tending to have limited political access and cultural capital, resulting in limited access to resources, land, and public health and social services. A land system perspective highlights that vulnerable individuals and communities tend to be also excluded from land use decisions and from knowledge and information networks (Asaaga et al., 2021, 2022). The largely unexplored synergies between One Health and environmental justice frameworks offer great potential to explicitly integrate a social dimension in understanding health risks (Murray et al., 2022).

Interventions to mitigate health risks linked to land systems must be prioritised and targeted towards the heterogeneous vulnerable groups and places to avoid further compromising livelihoods or adaptive capacity. Within the KFD case study, insights on social differentiation of vulnerability and adaptive capacity within the land system were gained by utilising participatory methods such as key-informant interviews, participant observations and household surveys to explore perceptions, livelihood and health priorities of affected communities. In Ecuador, the fraction of the production costs allocated to tick control is variable but larger for smaller farms (Paucar-Quishpe et al., 2023), suggesting that those with the most to lose from a worsening of the tick situation are the small farms operating on smaller budgets. In Scotland, the combination of size of holding, tenure regime and land control means that identifying a socially acceptable and just pattern of land use requires involving the community in framing and co-producing knowledge. This research would link health risks to land governance and land systems and integrate local knowledge and priorities into contextualised interventions for mitigating health risks.

Zooming in on land governance in our case studies reveals how long-term land use management resulting from decisions based on diverse actors and priorities have precipitated disease risks. These relationships between land system processes, land governance and health risks can be established robustly only by combining diverse disciplines and methodologies, and community engagement. Considering the long-term, distant interconnections between land use decision-making and health risks, land systems scientists have argued for new land use governance approaches that bridge diverse knowledge and value systems, reconnect actors to the consequences of their actions and recognise that local solutions to land systems challenges may only displace problems. For example, policies banning marginalised group from accessing the forests during KFD outbreaks or resettling them in new areas of forest (thus exposing them to new health risks), illustrate how top-down policy agendas, often rooted in one dominant value system (here National and State level forest preservation), can be contentious and counter-productive. In contrast, participatory approaches of co-production (Cornell et al., 2013) and community-based adaptation (Bardosh et al., 2017) hold the potential for reconciling diverse knowledge and value systems, understanding how trade-offs arise and co-developing integrated solutions for mitigating health risks such as contextualised risk communication (among socially differentiated groups) or risk-based surveillance and promotion of alternative livelihoods that reduce exposure, as was done for the case of KFD mitigation in India (Asaaga et al., 2022).

## Conclusion

Health consequences of land use have been a recurrent feature of human history – with many examples, e.g. in relation to domestication (Vourc’h et al., 2022). Zoonotic spill-overs have brought attention to the health collateral damages of land use changes, but by no means constitute the only health risk associated with land use. Many known environmentally driven human or animal health problems result from complex relationships between people, animals and land that have so far been examined over short time scales and often ignoring the broader context of land systems. Our three case studies demonstrate the value of integrating a land system perspective in disease ecology, namely a better understanding of health as resulting from land use legacies, of complex indirect or underlying drivers of land use and health dynamics, and of inequities in exposure and vulnerability, including in relation to control options, and the integration of land use/health trade-offs. Integrating health in land systems has the potential to identify new mitigation strategies compatible with livelihood priorities of vulnerable communities and with other priorities for land use, whether production or conservation. Health is therefore an integral part of land systems and should be an integral part of their governance.
